# Development of Folate Receptor−Targeted PET Radiopharmaceuticals for Tumor Imaging—A Bench-to-Bedside Journey

**DOI:** 10.3390/cancers12061508

**Published:** 2020-06-09

**Authors:** Silvan D. Boss, Simon Mensah Ametamey

**Affiliations:** 1 SWAN Isotopen AG, University Hospital Bern, 3010 Bern, Switzerland; silvanboss@hotmail.com; 2 Department of Chemistry and Applied Biosciences, Institute of Pharmaceutical Sciences, ETH Zurich, 8093 Zurich, Switzerland

**Keywords:** folate receptor, radiopharmaceuticals, PET imaging, structure-activity-relationship

## Abstract

The folate receptor-α (FR-α) is overexpressed in many epithelial cancers, including ovary, uterus, kidneys, breast, lung, colon and prostate carcinomas, but shows limited expression in normal tissues such as kidneys, salivary glands, choroid plexus and placenta. FR-α has therefore emerged as a promising target for the delivery of therapeutic and imaging agents to FR-positive tumors. A series of folate-based PET (positron emission tomography) radiopharmaceuticals have been developed for the selective targeting of FR-positive malignancies. This review provides an overview on the research progress made so far regarding the design, radiosynthesis and the utility of the folate-derived PET radioconjugates for targeting FR-positive tumors. For the most part, results from folate radioconjugates labeled with fluorine-18 (t_1/2_ = 109.8 min) and gallium-68 (t_1/2_ = 67.7 min) have been presented but folates labeled with “exotic” and new PET radionuclides such as copper-64 (t_1/2_ = 12.7 h), terbium-152 (t_1/2_ = 17.5 h), scandium-44 (t_1/2_ = 3.97 h), cobalt-55 (t_1/2_ = 17.5 h) and zirconium-89 (t_1/2_ = 78.4 h) are also discussed. For tumor imaging, none of the reported PET radiolabeled folates reported to date has made the complete bench-to-bedside journey except [^18^F]AzaFol, which made it to patients with metastatic ovarian and lung cancers in a multicenter first-in-human trial. In the near future, however, we expect more clinical trials with folate-based PET radiopharmaceuticals given the increasing clinical interest in imaging and the treatment of FR-related malignancies.

## 1. Introduction

### The Family of Folates and the Folate Receptor

**Figure 1:**
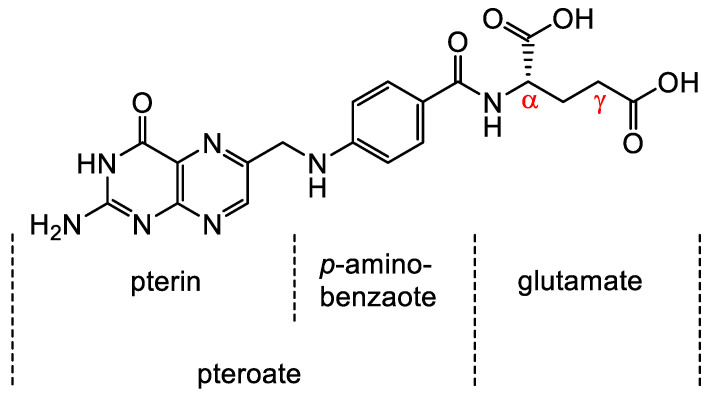
Chemical structure of folic acid (FA) consisting of glutamic acid entity and a pteroyl moiety that entails pterin and p-aminobenzoate groups. Derivatization of FA occurs via either the α- or γ-carboxyl group located in the glutamate moiety.

**Figure 2:**
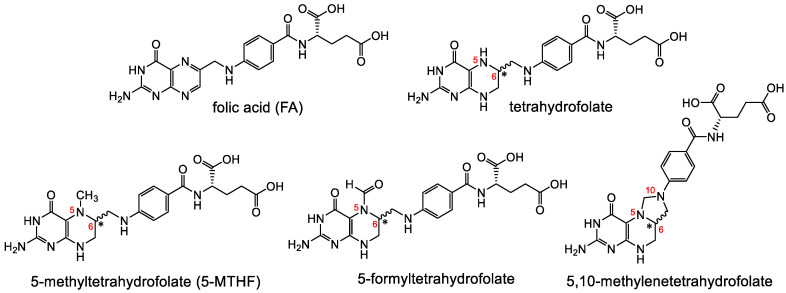
A selection of different forms of folates including the synthetic, fully oxidized FA and the endogenous reduced forms tetrahydrofolate, 5-MTHF, 5-formyltetrahydrofolate and 5,10-methylene-tetrahydrofolate. Reduced folates have a chiral center at position 6 of the pteroyl group.

Folates are hydrophilic molecules and belong to the B-group vitamins. Structurally, folates contain a glutamate entity and a pteroyl moiety that consists of pterin and p-aminobenzoate groups (Figure 1). They are crucial for normal cellular function as they are used for the one-carbon metabolic reaction and DNA biosynthesis, repair and methylation. Inadequate levels can result in severe health problems including cardiovascular diseases, anemia, embryonic developmental disorders and cancer. Folates have to be taken up from the diet as mammals are unable to synthesize folates by themselves [1,2]. The family of folates consists of several chemically different molecules including folic acid (FA) and a variety of reduced tetrahydrofolates (Figure 2). In case of the synthetic form of the vitamin, FA, the pterin group is fully oxidized. In vivo, however, the pteroate group is reduced resulting in tetrahydrofolate and/or several substituted derivatives. 5-Methyltetrahyrofolate (5-MTHF) is the predominant form found in blood and the most biologically active derivative. In contrast to FA, reduced folates have an additional chirality center at position 6 of the pteroate group (Figure 2) that gives rise to *S*- and *R*-stereoisomers, however, only the *S*-isomer is biologically active and present in mammals [2].

Mammals have three folate transporting systems: the reduced-folate carrier (RFC), the proton-coupled folate transporter (PCFT) and the folate receptor (FR) [3,4,5,6]. RFC is ubiquitously expressed in cells and represents the major transport system for folates from blood into the peripheral tissue, whereas the PCFT is mainly expressed in the liver and intestine and absorbs dietary folates and is responsible for in vivo folate homeostasis [7,8,9]. Four different isoforms of the FR have been reported in the literature to date [6]. The FR-γ and FR-δ are soluble proteins and found only in very low concentrations, whereas the expression levels of the FR-α and FR-β, which are both membrane-bound glycosyl-phosphatidylinositol-anchored membrane proteins, are high in particular organs in the body. FR-α is expressed in the proximal tubules of the kidneys, salivary glands, choroid plexus, placenta and the lung [10,11,12]. The FR-β expression is mainly in the placenta and hematopoietic cells of the myelogenous lineage [13]. As the FR-β is overexpressed on activated macrophages involved in inflammatory diseases, FR-β targeted imaging and therapy is considered as a promising application for radiolabeled folate conjugates [14,15].

The FR-α is overexpressed in many epithelial cancers including ovaries, uterus, kidneys, breast, lung, colon, prostate and brain. 90% of all ovarian and endometrial carcinomas and more than 70% of all lung and renal carcinomas express the FR at high levels. The overexpression of FR-α in cancer may have both cell growth regulation and signaling functions meaning that folate uptake can promote cancer cell proliferation, migration and loss of adhesion promoting cellular motility and metastasis. In addition, FR-α contributes to cancer malignancy by acting as a signaling molecule increasing cell motility and invasion.

Several studies have suggested that FR-α acts not only as folate transporter, but it also confers signaling and growth advantages on malignant cells [1]. As such, the FR-α has emerged as a promising target for the delivery of therapeutic and imaging agents to FR-positive tumors using folate conjugates [16,17,18,19,20,21]. FR-α binds to folate conjugates and mediates their internalization via the process of endocytosis. In this review, we report on folate-based radiopharmaceuticals for targeting FR using PET as an imaging modality. For the most part, results from folate radioconjugates labeled with fluorine-18 and gallium-68 are presented but folates labeled with “exotic” and new PET radionuclides such as copper-64, terbium-52, scandium-44, zirconium-89 and cobalt-55 are also discussed. In Table 1 is shown in chronological order the summary of the main findings of folate-based PET radiopharmaceuticals described in this review.

## 2. Structure-Activity-Relationship and Library Design of Radiolabeled Folate Derivatives

### 2.1. Chemical Modifications of Folic Acid to Produce Radiolabeled Derivatives

The crystal structure of human FR-α in complex with FA was determined by Chen et al. [64] in 2013 demonstrating that the pteroate moiety is buried inside the binding pocket of the FR, whereas the glutamate sticks out of the binding pocket entrance. Interestingly, they found that already small chemical modifications at the pterin group can result in a considerable loss of affinity. In contrast, derivatization of the α- and γ-carboxylic functionalities of the glutamate have no negative impact on the binding affinity to FR. Furthermore, the authors established that minor modifications of the phenyl group of the *p*-aminobenzoate group may be tolerated for retaining high binding affinity to the FR [64,65].

**Figure 3:**
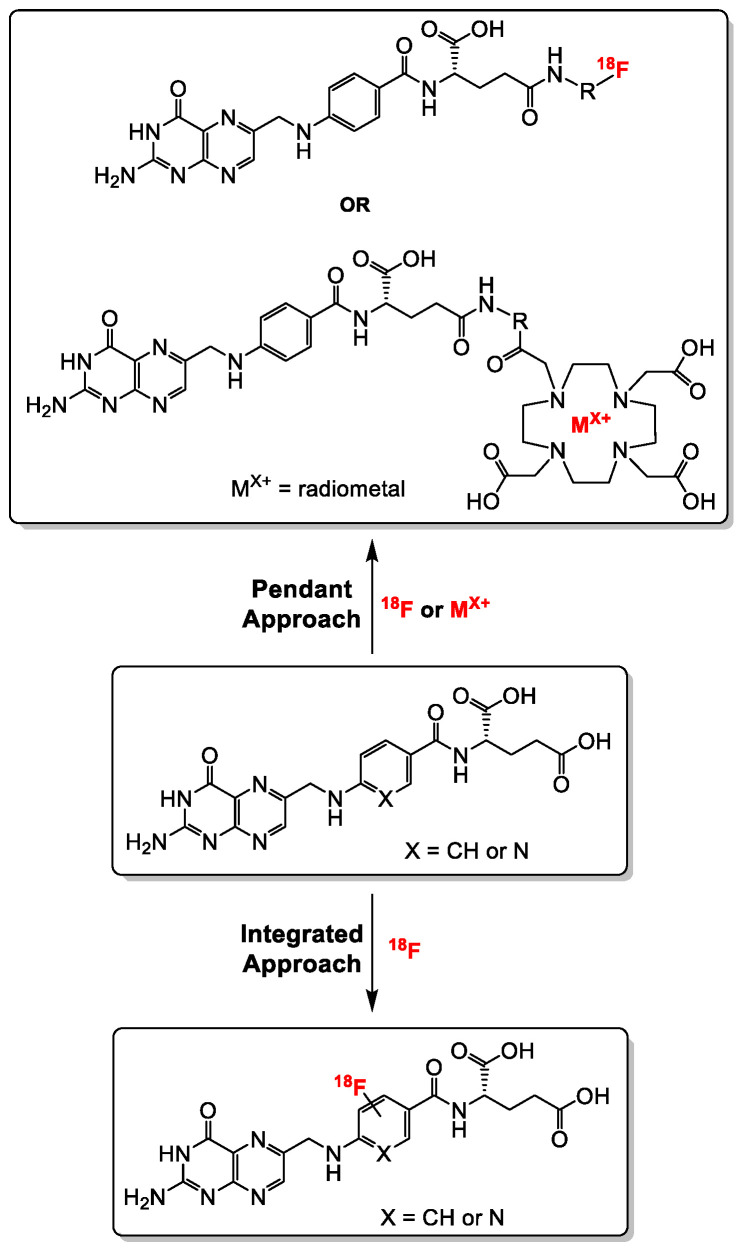
General synthetic strategies towards the synthesis of radiolabeled folates using the pendant and the integrated approaches. The radiolabeling of folates with radiometals is only possible via the pendant approach (DOTA chelator is shown as a representative example).

**Figure 4:**
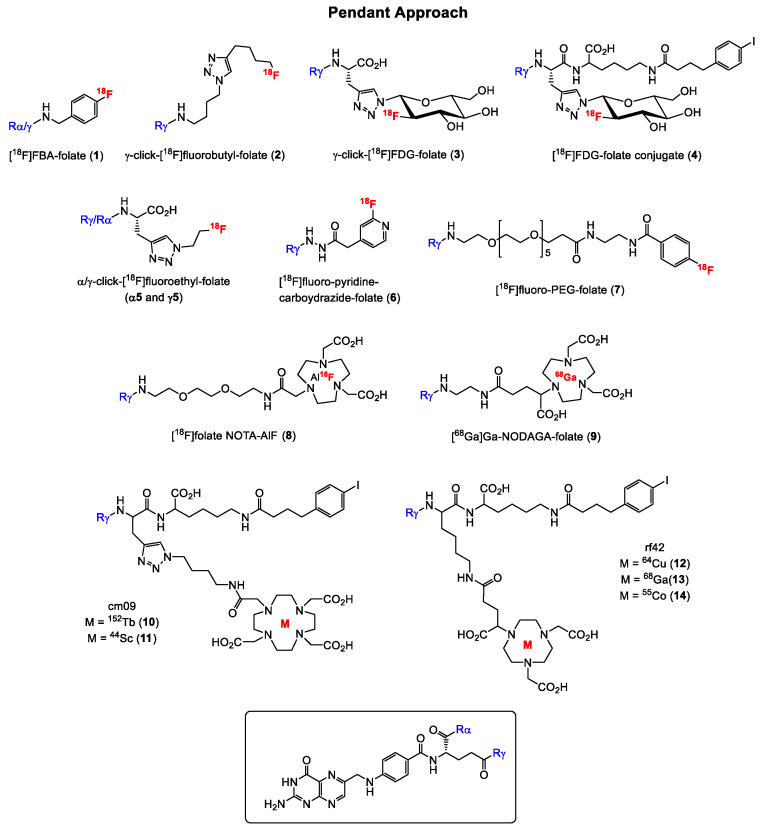
Structures of some notable and promising FA radiotracers prepared using pendant approach. For simplicity, radiometal coordination by chelators not shown.

**Figure 5:**
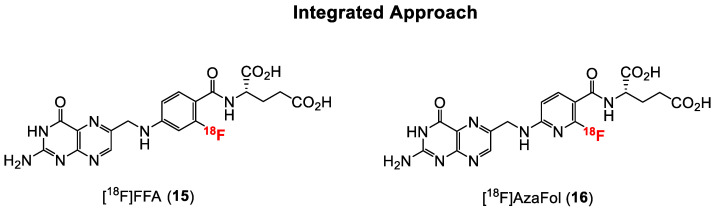
Structures of fluorine-18 labeled FA radiotracers prepared so far using integrated approach.

Generally, folate conjugates bearing either chemotherapeutics for therapeutic or prosthetic groups for imaging purposes have been synthesized using the so called “pendant approach”, whereby their conjugation to FA proceeds via one of the two carboxylic functionalities at either the alpha (α) or gamma (γ) position of the glutamate moiety (Figure 3). Another derivatization method termed “integrated approach” which will be explained later, has also been applied for the radiolabeling of folates. In Figure 3 are shown the general structures of radiolabeled folates derived from either the pendant or integrated approach. Almost all folic acid-based chemotherapeutic drugs and radiopharmaceuticals have been prepared using the pendant approach via derivatization at the γ-position. This can be explained by the fact that the α-carboxylic acid group in the glutamate part of FA is synthetically less easily accessible for conjugation due to steric hindrance compared to the γ-carboxylic acid group [15,66]. Depicted in Figure 4 and Figure 5 are some examples of radiolabeled folate derivatives prepared using either the pendant or integrated approach.

#### 2.1.1. α-and γ-Derivatization of the Glutamate Groups in Folic Acid—The Pendant Approach

A variety of radiolabeled folate derivatives for single photon emission computed tomography (SPECT) using the pendant approach have been published with the first report in 1994 describing a folic acid conjugate radiolabeled with iodine-125 [67,68,69,70]. The first FR-targeted radiopharmaceutical for PET imaging was reported 9 years later in 2003 by Mathias and co-workers [22] who radiolabeled deferoxamine-folate with two positron emitting isotopes of gallium, gallium-66 (half-life = 9.5 h) and gallium-68 (half-life = 68 min). The radiosynthesis was performed in analogy to the method described in the literature for the SPECT [^67^Ga]Ga-radiolabeled analogue [71], whereby the utility of only the [^66^Ga]Ga-labeled folate derivative for targeting FR-positive tumors using KB tumor-bearing was subsequently investigated. KB cell line is a cervical cancer cell line that expresses the FR at high levels and is therefore used as the “gold standard” for FR-targeting in in vitro and in vivo studies [72]. For the radiolabeling with ^66^Ga and ^68^Ga, a mixture of both the α- and γ-isomers of the folate derivatives were used, and high radiochemical purities were obtained for both folate conjugates. Studies in FR-positive KB tumor-bearing mice with [^66^Ga]Ga-deferoxamine-folate revealed a heterogeneous tumor uptake, which was blocked by co-injection of folic acid, indicating specific binding to the tumor. A shortcoming of [^66^Ga]Ga-deferoxamine-folate, however, was the high kidney and abdominal uptake in addition to the poor PET image qualities resulting from the unfavorable physical properties of ^66^Ga. ^66^Ga has a maximum positron energy of 4.15 MeV. For comparison, the most widely used positron emitter, ^18^F, has a maximum positron energy of 0.63 MeV, which is factor 6.5-fold less. In 2005, Rossin et al. [23] reported on ^64^Cu-labeled folate-conjugated shell cross-linked nanoparticles for tumor imaging. The shell cross-linked nanoparticles (SCKs) were functionalized with folate, fluorescein thiosemicarbazide and a TETA (1,4,8,11-tetraazacyclotetradecane-N,N′,N″,N‴-tetraacetic acid) chelator for labeling with ^64^Cu. In vivo studies with ^64^Cu-TETA-SCK-folate revealed a KB tumor uptake of 5.9 ± 2.8% injected activity per gram (IA/g) at 4 h p.i. (post injection). Blocking experiments with excess folic acid showed reduced accumulation of ^64^Cu-TETA-SCK, suggesting specific accumulation of the folate derivative in FR-positive KB tumors.

The past fifteen years, however, have witnessed a surge in the development of fluorine-18 labeled folate radioconjugates for FR imaging mainly due to the increased availability of PET scanners. The first ^18^F-labeled FA radiotracer designated 4-[^18^F]fluorobenzylamine-folate ([^18^F]FBA-folate, **1**, Figure 4) was published by Ametamey and co-workers in 2006 [24]. The pendant approach was used to conjugate 4-[^18^F]FBA to FA non-selectively resulting in a 1:4 mixture of the α- and γ-conjugated radiolabeled folates, respectively. These results demonstrated that the γ-position is more easily accessible for conjugation compared to the α-position of the glutamate. Besides the non-selective radiosynthesis, a major drawback of **1** was the high abdominal uptake observed during the PET imaging studies. In order to circumvent the formation of a mixture of the α- and γ-conjugated radiolabeled folates, the same research group performed a regiospecific radiosynthesis of diastereomerically pure γ-click-[^18^F]fluorobutyl-folate (**2**, Figure 4) using the γ-conjugated folate precursor obtained via a stereoselective organic synthesis.

During the organic synthesis of the precursor, α-protected glutamic acid was used [25,73]. Compound **2** was obtained by a Cu(I)-catalyzed azide-alkyne cycloaddition in high radiochemical yields of 25–35% and high molar activities of 160 ± 70 GBq/µmol. The binding affinity of the γ-folate conjugate was determined to be 18.0 ± 7.1 nM, however, a tumor uptake of only 3.13 ± 0.83% (IA/g) at 45 min p.i. was found in KB tumor-bearing mice. High uptake of **2** was observed in the abdominal region and the bile due to the high lipophilicity of the radiotracer caused by the fluorobutyl moiety. The in vivo results of both tracers **1** and **2** indicated that high lipophilicity of folate conjugates results in a high uptake in the gastrointestinal tract and thus, an unfavorable in vivo distribution profile. Based on these observations, Ametamey and co-workers [31] in 2012 synthesized and investigated a more hydrophilic folic acid-based radiotracer designated γ-[^18^F]fluoro-deoxyglucose folate (γ-[18]FDG-folate, **3**, Figure 4). As expected, the hydrophilic glucose entity caused a considerable change in the polarity of **3** compared to derivatives **1** and **2** resulting in an increased tumor uptake of 10.03 ± 1.12% IA/g 60 min p.i. in FR-positive KB tumor-bearing mice. Nevertheless, a high liver uptake was still observed and as expected a very high uptake in FR-positive kidneys (42.94 ± 2.04% IA/g 60 min p.i.) was also evident. Although the kidney is a FR-positive organ, the high retention observed could not only be attributed to binding to FR in the kidneys but also to unfavorable pharmacokinetics. Previous studies have demonstrated that the incorporation of an albumin-binding entity into radioconjugates resulted in reduced kidney uptake due to increased circulation time in the blood [74,75]. This strategy was applied wherein **3** was modified and conjugated to such an entity [34]. The new γ-[18]FDG-folate conjugate **4** bearing an albumin-binding entity exhibited a four-fold reduced kidney uptake compared to **3** due to the enhanced blood circulation time. High tumor uptake and an excellent tumor-to-kidney ratio could be demonstrated. However, a major drawback of **4** was the low radiochemical yield of only 1–2% (decay corrected).

In order to investigate the impact of α- and γ-conjugation of radiolabeled folates on their biological behavior, an extensive study was performed by Boss et al. in 2016 [43]. Three pairs of fluorinated α- and γ-conjugated FA derivatives, namely α- and γ-click-fluorobutyl-folates (α-isomer in analogy to **2**), α- and γ-click-fluoroethyl-folates (**α5** and **γ5**) and the α- and γ-click-FDG-folates (α-isomer in analogy to **3**) depicted in Figure 4, were prepared and biologically evaluated with the specific aim to assess whether differences exist in the in vitro and in vivo characteristics of the regioisomers. The results showed that the site of conjugation in FA has an impact on the in vivo biodistribution profile of ^18^F-labeled folate conjugates but not on the in vitro binding affinity to FR. All the folate derivatives showed a single-digit nanomolar binding affinity (IC_50_ (half maximal inhibitory concentration) = 1.4–2.2 nM) to FR. Generally, the α-isomer of all the three pairs of folate conjugates exhibited a significantly increased kidney uptake, whereas the corresponding γ-regioisomers showed a significantly higher liver uptake. The authors speculated that unspecific carrier-mediated uptake via organic anion transporters or PCFT could be responsible for the difference in the liver uptake of the regioisomers [43].

In 2006, Al Jammaz et al. [26] reported a three-step radiosynthesis of [^18^F]fluoropyridinecarbohydrazide-folate (**6**) for targeting the FR, however, in this report no biological data was provided. A few years later, the same group published fluorine-18 labeled pyridinecarbohydrazide-methotrexate conjugates as well as an [^18^F]FDG-folate derivative and reported biological data of all the derivatives [27,32]. Absolute tumor uptake values of all these tracers did not exceed 6% IA/g, and rapid blood clearance with excretion by the urinary pathway was observed. Similar results in terms of high kidney uptake and in vivo behavior were obtained with [^18^F]oligoethyleneglycol (OEG)-folate and a ^18^F-labeled folate-pHPMA conjugate reported by Schieferstein et al. [36,38]. Another [^18^F]fluoro-polyethylene glycol (PEG)-folate (**7**, Figure 4) was reported by Gent et al. [33] in 2013 with the aim to image activated macrophages in a rat model of arthritis. A first-in-human study was recently performed with this tracer in rheumatoid arthritis patients demonstrating a high potential for imaging inflammatory activity in the whole body [76]. Other folate derivatives labeled with ^18^F and ^68^Ga have been published by Kularatne et al. [35] with the aim to image activated macrophages in an inflammatory paw model. The PET imaging of inflammatory activity in patients suffering from inflammatory diseases seems to be another promising area of application for radiolabeled folates, however, this topic is not further discussed here as the focus of this review is mainly on tumor imaging.

A few years ago in 2016, Chen et al. [42] reported on [^18^F]folate-NOTA-AlF (**8**, Figure 4). The radiosynthesis was accomplished by reacting [^18^F]fluoride with AlCl_3_ for 2 min followed by heating with folate-NOTA at 100 ^ο^C for 15 min. Compund **8** was obtained in 18% radiochemical yield and a molar activity of 68 GBq/µmol. FR binding studies revealed a K_d_ (dissociation constant) value of 1 nM. In vivo PET imaging and ex vivo biodistribution studies demonstrated high and specific binding to FR-positive tumors. However, the liver uptake of **8** was not optimal. To circumvent this shortcoming, the same group later introduced a PEG_12_ unit instead of PEG_1_ into [^18^F]folate-NOTA-AlF in order to increase its hydrophilicity [45]. The radiosynthesis of the new radioconjugate, [^18^F]folate-PEG-NOTA-AlF was achieved using the procedure reported for the non-PEGylated radiolabeled folate. In PET imaging and ex vivo biodistribution studies, a two-fold less liver uptake was observed when compared to **8**, suggesting that the increased hydrophilicity caused an improvement in the pharmacokinetic behavior of the PEGylated folate derivative [45]. In 2018, Kettenbach and co-workers [47] published the radiosynthesis and biological evaluation of two new ^18^F-labeled folate conjugates, designated [^18^F]DBCO- and [^18^F]Ala-folate. In biodistribution and PET imaging studies, both tracers showed low tumor uptake of only 0.5–1.6% IA/g, however, high kidney and abdominal uptake was observed, which could be explained by the increased lipophilicity of the tracers.

Besides fluorine-18, an increasing number of positron emitting radiometals such as ^68^Ga (maximum energy of emitted β^+^ particle (β^+^_max_) = 1.90 MeV, t_1/2_ = 67.7 min), ^64^Cu (β^+^_max_ = 0.65 MeV, t_1/2_ = 12.7 h), ^152^Tb (average energy of emitted β^+^ particle (β^+^_ave_) = 1.08 MeV, t_1/2_ = 17.5 h), ^44^Sc (β^+^_max_ = 1.47 MeV, t_1/2_ = 3.97 h), ^55^Co (β^+^_max_ = 1.50 MeV, t_1/2_ = 17.5 h) and ^89^Zr (β^+^_max_ = 0.89 MeV, t_1/2_ = 78.4 h) have been employed more recently for the radiosynthesis of folate conjugates. Prominent among them is ^68^Ga given its easy accessibility via the ^68^Ge/^68^Ga generator system and a broad application in the clinic. In 2011, Fani et al. [28] published a ^68^Ga-labeled DOTA folate conjugate for FR-positive tumor imaging. High tumor uptake of 12% IA/g was obtained after 1h p.i.. This radiolabeled folate showed improved pharmacokinetics compared to the previously reported ^66/67/68^Ga-deferoxamine-folate. One year later, the same group reported on ^68^Ga-labeled NODAGA-folate (**9**, Figure 4) and 5,8-dideazafolic acid derivative, which was used as a pharmacophore based on the structure of the first clinically evaluated folate-based thymidylate synthase inhibitor [29]. The folate derivative **9** outperformed the 5,8-dideazafolic acid derivative and the previously reported [^68^Ga]Ga-DOTA-folate showing a high tumor uptake of around 16% IA/g already 1h p.i., fast blood clearance and low hepatobiliary uptake. However, high kidney uptake resulting in a low tumor-to-kidney ratio was found. In 2013, Kim et al. [77] reported on an interesting folate-based imaging agent composed of the inhibitor of metalloproteinase-2 (TIMP-2), human serum albumin, NOTA chelator and folic acid [77]. By targeting malignant FR-positive tissues using FA, TIMP-2, which exhibits antitumor activity through its antiangiogenic properties, may have a therapeutic effect on the cancer tissues. For tracking, the so called HT2-folate was radiolabeled with ^68^Ga. In biodistribution studies with KB xenograft-bearing nude mice, a tumor uptake of only 2% IA/g at 1 h p.i. and high liver and spleen uptake were observed. High abdominal uptake and weak uptake in the KB tumor was also evident in the PET imaging studies. Al Jammaz et al. [39] published results on ^68^Ga-labeled NOTA- and NOTAM-folate conjugates and found high tumor uptake, but also moderate uptake in the kidneys and liver for both folate conjugates. Another ^68^Ga-labeled NOTA-folate bearing a PEG-entity was prepared by Brand et al. [44] and the radiolabeled folate was compared to [^99m^Tc]Tc-EC20 (Etarfolatide), which represents the most known SPECT imaging agent for FR targeting that has been evaluated in several clinical trials [78,79,80,81,82]. Comparable results were obtained for both ^68^Ga-NOTA-folate and [^99m^Tc]Tc-EC20. Choi et al. [48] prepared [^68^Ga]Ga-HBED-CC-EDBE-folate, which showed no favorable in vivo characteristics.

Besides ^68^Ga, a small number of publications are available on other positron emitting radiometals including ^152^Tb, ^44^Sc, ^64^Cu, ^55^Co and ^89^Zr, which are currently not widely used due to their restricted availability but are under investigation for potential consideration for future application. In 2012, Müller et al. [30] reported for the first time a proof-of-concept study with terbium-labeled folate derivatives. The precursor cm09 bearing a DOTA-chelator was radiolabeled with ^161^Tb, ^149^Tb, ^152^Tb or ^155^Tb in radiochemical yields of >96%. ^152^Tb is a β^+^-emitter and [^152^Tb]Tb-cm09 (**10**, Figure 4) was therefore used for PET imaging with FR-positive KB tumor-bearing mice. Due to the high positron energy of ^152^Tb, relatively poor spatial resolution of the PET images were obtained. However, 24 h after injection, the tumor xenografts and kidneys were clearly visualized. The first scandium-44-labeled DOTA-folate conjugate, namely [^44^Sc]Sc-cm09 (**11**, Figure 4) was reported by Müller et al. [37]. Excellent tissue distribution was observed for [^44^Sc]Sc-cm09 with high radioactivity values in FR-positive tumor of 8% IA/g and 14% IA/g at 2 h and 20 h p.i., respectively and radioactivity uptake in non-targeted tissues such as liver, muscle and intestinal tract was very low. Farkas et al. [40] reported on a folate conjugate bearing an albumin-binding entity to increase the circulation time of the radiolabeled folate and a NODAGA-chelator for labeling with ^64^Cu and ^68^Ga for PET imaging of FR-expressing tumor-bearing mice. High tumor uptake of 15 and 12% IA/g was observed 4 h p.i. for [^64^Cu]Cu-rf42 (**12**, Figure 4) and [^68^Ga]Ga-rf42 (**13**, Figure 4), respectively. The NODAGA-chelator seemed to be more suitable for a stable coordination to ^64^Cu compared to the DOTA-chelator. A NOTA-folic acid conjugate radiolabeled with ^68^Ga was prepared by Jain et al. [41]. In vitro cell binding studies showed specificity of the folate derivative to FR, however, no results of in vivo studies with FR-positive tumor-bearing mice have been reported. In 2019, Radford et al. [49] reported the radiolabeling of folate conjugates rf42 and cm10 with ^55^Co for PET imaging representing the first cobalt-55-labeled radiolabeled folates ever to be reported in the literature. A high radiochemical yield of ≥95% and in vitro stability of ≥93% of both tracers were obtained. In addition, a high KB tumor uptake of 17% IA/g at 4 h p.i. was found, however, the tumor-to-kidney uptake was not improved compared to the corresponding previously reported ^64^Cu- and ^68^Ga-radiolabled radiolabeled folates. In contrast, the ^55^Co-labeled folate conjugate (**14**, Figure 4) exhibited lower liver uptake compared to the ^64^Cu-labeled radiolabeled folates. This observation was explained by the fact that ^55^Co in contrast to ^64^Cu does not accumulate in the liver when it is released by the DOTA or NODAGA chelator since it behaves as a calcium mimetic [49].

In 2019, Heo et al. [50] reported on a FR-targeting agent composed of the FR-α-binding humanized monoclonal antibody M9346A labeled with ^89^Zr with the aim to evaluate FR-α expression in triple-negative breast cancer patients and to guide intervention with the therapeutic antibody-drug conjugate IMGN853 that is currently under evaluation in multiple clinical trials. ^89^Zr-M9346A revealed promising biological characteristics for the mentioned purposes, however, according to the authors, further studies needed to be carried out.

Multifunctional folic acid-modified dendrimers labeled with ^64^Cu and using a DOTA chelator has also been reported by Ma et al. [46], however, high liver and kidney uptake and only moderate KB tumor uptake was obtained. Folate-conjugated iron oxide nanoparticles radiolabeled with either ^64^Cu or ^68^Ga for imaging purposes have been prepared and biologically evaluated in cell studies showing high specific interaction between FA on the nanoparticles and the FR of the FR-positive cells [51,52]. The in vivo performance of these radiolabeled folate-conjugated nanoparticles has not been reported and remains to be investigated.

Several radiolabeled pteroyl-conjugates have been reported in the literature, however, no favorable in vivo characteristics compared to folate conjugates were found for these derivatives. Low tumor and high kidney uptake were observed, suggesting that the glutamate moiety plays an important role in the biodistribution of the folate derivatives [53,54,55,56,57].

#### 2.1.2. Direct Labeling of Folate Backbone—The Integrated Approach

All the above-mentioned radiofolic acid conjugates were prepared via the pendant approach. Another approach, as mentioned earlier (Figure 3), is the integrated approach, wherein the [^18^F]fluoride ion is directly installed onto the folate backbone without the need for a chelator or prosthetic group.

The first folic acid derivative obtained via the integrated approach was reported in 2010 by Ametamey and co-workers [58]. The 2^′^-[^18^F]fluorofolic acid derivative ([^18^F]FFA, **15**, Figure 5) was obtained via a two-step radiosynthesis involving a nucleophilic aromatic substitution of a nitro group, followed by acidic removal of the protecting groups. A major disadvantage of this folate derivative was the low overall radiochemical yield of only 4%, which is suboptimal for potential clinical application. The recently established Cu-mediated ^18^F-fluorination radiolabeling method of non-activated aromatic systems from pinacol boronate esters, boronic acids or stannanes precursors would certainly afford a higher overall radiochemical yield [83]. Competitive in vitro cell binding studies with **15** showed a similar low nanomolar binding affinity when compared to native folic acid, indicating that minor modifications on the pteroyl moiety of folic acid do not have a negative impact on the in vitro binding affinity to the FR. In in vivo studies, a similar high tumor uptake value of around 10% IA/g compared to the previously mentioned click-[^18^F]FDG- (**3**) and click-[^18^F]fluoroethyl-folates (**5**) was found for [^18^F]FFA. Abdominal, kidney, liver and intestine uptake of [^18^F]FFA was evident in the PET images.

Facilitated ^18^F-labeling via nucleophilic aromatic substitution can be accomplished by the use of *ortho*- or *para*-substituted heteroaromatic rings such as pyridines [84]. As such, the nucleophilic aromatic substitution at the *ortho* position of a less electron rich pyridine incorporated into the core structure of folic acid might afford a higher overall radiochemical yield without the need for activating groups on the pyridine ring. Consequently, Ametamey and co-workers [59] designed and synthesized a new folate derivative wherein the phenyl ring in the pteroyl moiety was replaced by a pyridine ring, which resulted in an aza-folic acid derivative named 3′-aza-2′-[^18^F]fluorofolic acid ([^18^F]AzaFol, **16**, Figure 5). [^18^F]AzaFol exhibited an in vitro binding affinity (IC_50_) value of 0.8 ± 0.2 nM to FR. This binding affinity value is similar to that of native folic acid (1.1 ± 0.4 nM) and suggests that the replacement of carbon atom with a nitrogen atom in the phenyl moiety of the folic acid has no detrimental effects on the in vitro binding affinity to FR. Indeed, and as anticipated, the radiosynthesis of **16** could be accomplished in a decay corrected radiochemical yield of 3–9% over two steps, which was higher compared to **15**. In addition, **16** showed a higher tumor uptake of 12.6 ± 1.77% IA/g at 90 min p.i. compared to **15**. Uptake of [^18^F]AzaFol in FR-positive kidney and the liver was comparable to the uptake values of [^18^F]FFA. From these radiochemical and biological results, it can be concluded that compared to [^18^F]FFA, [^18^F]AzaFol is more suitable for transferring to an automated synthesis module for possible translation to the clinic.

### 2.2. Reduced Folate Derivatives

**Figure 6:**
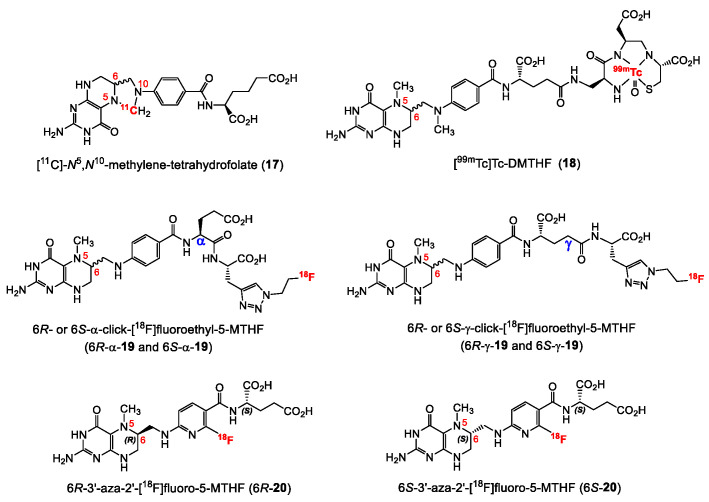
Structures of radiolabeled reduced folate radiotracers.

Until recently, the development of folate radiopharmaceuticals mainly focused on folic acid-based derivatives, and no alternative folate-derived structure was considered. This may be explained by the higher synthetic accessibility, the greater stability and the higher binding affinity towards FR of the oxidized form of FA derivatives compared to reduced folates. Importantly, reduced folates exhibit an interesting characteristic by showing subtype selectivity for the FR-α over FR-β. The FR-β isoform is overexpressed mainly on activated macrophages involved in inflammations. Selective tumor over inflammation imaging would therefore be possible with reduced folate radiopharmaceuticals. A selection of radiolabeled reduced folate derivatives reported in the literature is shown in Figure 6. The first reduced folate derivative radiolabeled with a positron emitting radionuclide was a ^11^C-labeled compound ([^11^C]-*N^5^*,*N^10^*-methylene-tetrahydrofolate, **17**, Figure 6) reported in 2012, however, neither information regarding the diastereomeric ratio of the radiolabeled product nor biological results were reported [60]. Although not a PET radiotracer, [^99m^Tc]Tc-DMTHF (**18**, Figure 6), a dimethyltetrahydrofolate derivative reported by Vaitiligam et al. [61] is the only reported reduced folate labeled with a SPECT radioisotope. Selectivity studies for the FR-α over FR-β were carried out in vivo showing a selective targeting of FR-α over FR-β thereby exhibiting the potential to distinguish cancer from inflammation. However, to date no further results on **18** have been published. The first fluorine-18 labeled 5-MTHF PET tracer was reported by Boss et al. in 2018 [62]. Four isomers of ^18^F-labeled click-fluoroethyl-5-MTHF derivatives (**19**, Figure 6) were synthesized using the pendant approach and evaluated for their utility as FR-targeting radiopharmaceuticals. Radiochemical purities above 95% were obtained for the four radioligands after the addition of a cocktail of antioxidants that included sodium ascorbate and L-cysteine. The four isomers exhibited similar in vitro binding affinities (IC_50_ = 17.7–24.0 nM) to FR-α. Comparable high tumor uptake values (8–11% IA/g) were found for the reduced folates compared to the FA analogues despite the lower binding affinity of the 5-MTHF derivatives. Interestingly, the *R*- and *S*-isomers exhibited different absolute uptake values in various organs including the kidneys and the liver, however, no significant changes in the uptake were found for both the α- and γ-conjugated diastereoisomers. This was explained by the different pharmacokinetics of the *R*- and *S*-isomers, which show different affinities to transport systems such as the PCFT. The authors concluded that further experiments needed to be done in order to confirm this pharmacokinetic behavior and also experiments to investigate their selectivity for FR-α over FR-β. Nevertheless, this study demonstrated that radiolabeled reduced folates represent a promising alternative to FA for targeting FR-positive cancer tissues.

**Figure 7:**
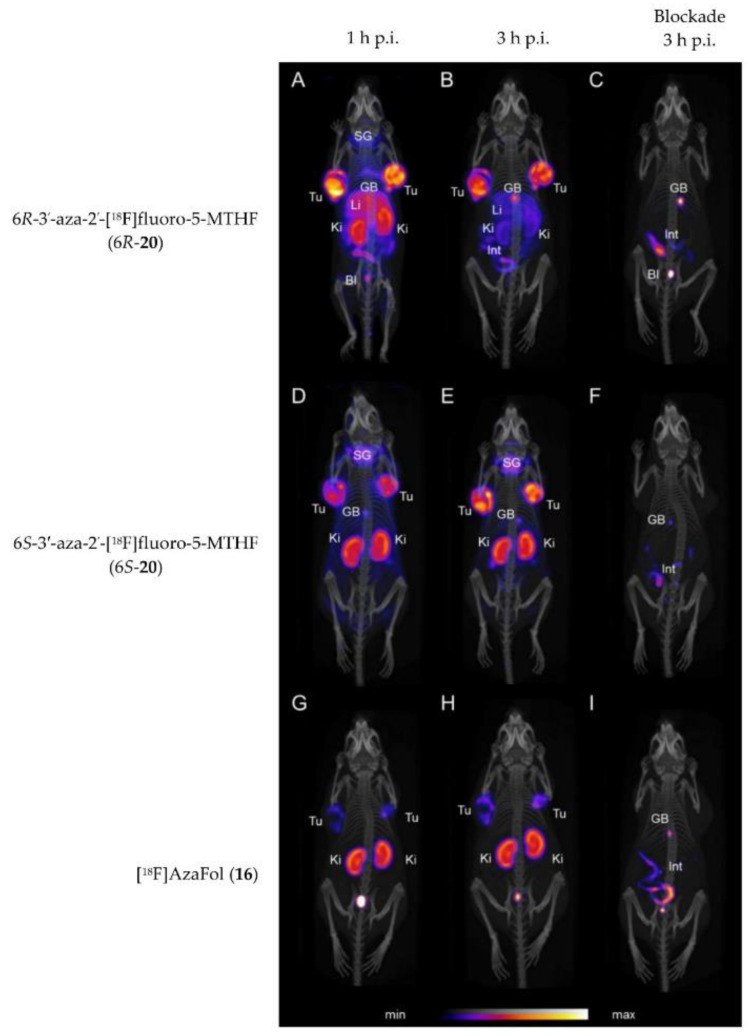
PET/CT images of KB tumor-bearing mice at 1 and 3 h p.i. and treatment with folic acid at 3 h p.i. of 6*R*-**20** (**A**–**C**), 6*S*-**20** (**D**–**F**) and **16** (**G**–**I**). Tu = KB tumor; Li = liver; Ki = kidney; SG = salivary glands; GB = gall bladder; BI = urinary bladder; Int = intestines/feces. This figure was adapted from Boss et al. [63].

Very recently, in 2019 Boss et al. [63] reported on another series of 5-MTHF radiotracers. The integrated approach was used to prepare the 6*R*- and 6*S*-3′-aza-2′-[^18^F]fluoro-5-MTHF derivatives (6*R*-**20** and 6*S*-**20**, Figure 6), which are the corresponding reduced forms of the previously described [^18^F]AzaFol (**16**, Figure 5). 6*R*- and 6*S*-**20** were obtained in a decay-corrected radiochemical yield of up to 5%, which was lower compared to the FA radiotracer **16** (9%). An explanation for this low radiochemical yield is the lower chemical stability of 5-MTHF compared to FA. Both 5-MTHF isomers exhibited lower and similar binding affinities to the FR-α (27 and 24 nM) compared to the oxidized form [^18^F]AzaFol **16** (1.4 nM). Biodistribution studies, however, revealed a high unprecedented tumor uptake of over 32% IA/g for both 6*R*- and 6*S*-**20** at 3 h p.i., whereas for the corresponding oxidized FA derivative **16** only 15% IA/g was found in the KB tumors. Similar to the findings of the *R*- and *S*-click-[^18^F]fluoroethyl-5-MTHF isomers (**19**), different radioactivity uptake of the 6*R*-**20** and 6*S*-**20** in the kidneys, liver, salivary glands and spleen was also observed. The authors suggested that different transport mechanisms of the three aza-folates (**16**, 6*R*-**20** and 6*S*-**20**) by the folate transporting systems might be a reason for the observed different biological behavior. In general, the reduced radiolabeled folates outperformed the corresponding oxidized form in terms of tumor visualization in PET images (Figure 7).

## 3. Selecting Lead Candidates for Further Investigation Leading to Clinical Studies

Radiotracer development is an iterative process consisting of several consecutive steps starting with the definition of a clinical need. In principle, the development process for imaging agents is very similar to that of drug development but can sometimes be more challenging. Once a clinical need is defined, a suitable target with altered expression relevant for imaging and treatment is identified. In a next step, appropriate target compounds or lead structures that efficiently bind to the target are designed and synthesized. Generally, an in vitro screening to find the most promising candidate from the synthesized library is carried out, after which the radiosynthesis of the most promising molecule is developed and established. The radiosynthesis should be as straightforward and high yielding as possible and result in a radioproduct with a radiochemical purity of >95%.

In addition, the radionuclide must be easily accessible, affordable and available in sufficient amounts. The radiotracer should be extensively evaluated in in vitro and in vivo experiments to elaborate its biological characteristics. In case of appropriate results, a proof-of-concept study in patients may be performed. An optimization of the radiosynthesis shall be carried out before the translation of the radiolabeled folate production to a Good Manufacturing Practice (GMP) facility in order to guarantee a sufficient amount of product for the patients.

Finding a promising lead candidate for further investigation leading to clinical studies requires profound knowledge and a line of research and development in the fields of target identification, organic chemistry, radiochemistry, pharmacology and medical needs [85]. Key parameters to consider are high affinity and selectivity to the target, which must be high, choice of radionuclide, site of radiolabel, clearance and metabolism. The most important aspect for tumor imaging is to bring as much radioactivity as possible to the malignant tissue in order to visualize the cancer tissue, whereas benign tissue and organs should not interfere with or mask the malignant tissue by a high uptake of radioactivity. For radiolabeled folates, it is worth mentioning that since the FR-α is expressed also in the kidneys, considerable high radioactivity uptake is normally observed in this organ. As a consequence, radiolabeled folates generally exhibit a low tumor-to-kidney ratio, which is critical for therapeutic purposes. For imaging purposes, however, high tracer uptake in the kidneys is not so much an issue. Nevertheless, dosimetry studies of folate radiopharmaceuticals entering clinical studies must be performed in order to estimate the absorbed dose per organ and the whole body absorbed dose (effective dose) per examination. This information is also relevant for the local health authorities, licensing and controlling bodies.

Based on the research performed during the last decades, it became evident that the (radio)chemistry behind folate derivatives is challenging and not straightforward especially in the case of reduced folates. Reduced folates have decreased chemical stability and are more sensitive to oxidation than the fully oxidized FA, and therefore induce additional challenges during their radiosynthesis. Generally, ^18^F-labeled folate derivatives are obtained in low to moderate radiochemical yields and HPLC (high-performance liquid chromatography) purification of the final product is required to achieve a radiochemical purity of >95%. In contrast, radiolabeling of folate conjugates with radiometals normally affords the final product in high radiochemical yields and purity after extensive optimization of the labeling conditions.

For preclinical investigations (in vitro and in vivo) of radiolabeled folate derivatives, cervical FR-expressing KB tumor cell lines and xenografts have evolved as the standard model. Nonetheless, tumor models involving other cell lines such as IGROV-1 (ovarian), SKOV-3.ip (metastases-like tumors) or SKBR3 (breast) can be employed [72]. These cell lines can also be used to determine the in vitro binding affinities and the selectivity of the folates to both the FR-α- and FR-β isoforms after stably transfection with the respective isoforms. A further evaluation in in vivo studies can then be undertaken if a high enough binding affinity and high specific cell uptake in FR-positive cells can to be demonstrated. Selectivity to either the FR-α- and FR-β isoforms may also be evaluated in vivo using mice models having both a FR-α-positive xenograft and an induced inflammation by e.g., injecting a cardiotoxin into muscle of mice or by adding dextran sulfate sodium to the drinking water of mice as reported by Vaitilingam et al. [61]. Although, KB tumor-bearing mice has evolved as the standard, and most results on the pharmacokinetics, biodistribution and the tumor imaging of the folate radiotracers have been obtained using this mice model, other mice xenograft models using other FR-expressing cell lines mentioned above potentially could be used depending on the research topic.

Several cut off criteria exist for the selection of radiolabeled folates for clinical translation. First, high binding affinity to the FR is a necessary criterion. The results obtained for the derivatives 6*R*-**20** and 6*S*-**20**, which showed an IC_50_ value of around 25 nM and exhibited a tumor uptake of >32% IA/g at 3 h p.i., suggest that tan IC_50_ value as high as 30 nM may be sufficient for the in vivo imaging of the FR. Second, the lipophilicity (logD value) of folate derivatives seems to be an important parameter since data in the literature suggests that folate derivatives with logD values less than −3 generally show more favorable biological characteristics compared to radiotracers with logD values greater than −3. Third, the size (molecular weight) of the folate derivative seems to be of less importance for a promising biological performance as favorable results were obtained with compounds ranging from ~500–2000 g/mol. Finally, for derivatives using the pendant approach, the site of modification at the glutamate moiety should be considered carefully especially for the oxidized forms since in vivo biological characteristics may differ for the different regioisomers.

Based on preclinical results obtained so far from the many different radiolabeled folate derivatives reported, it has become clear that high tumor uptake and good visualization of FR-expressing tumor tissue in mice can be achieved within the first hours since tumor uptake of so far reported radiolabeled folates did not considerably increase beyond a period of 2–3 h. For this reason, radionuclides such as ^68^Ga and ^18^F and potentially also ^44^Sc with a half-lives of more than one hour are potentially suitable for imaging FR-positive tumors.

## 4. Important Considerations for Translating Folate Radiopharmaceuticals for Human Studies

Once a candidate with promising attributes from the preclinical study has been identified, a proof-of-concept study in humans can be performed. Several important considerations for translating a folate radiopharmaceutical for human studies have to be taken into account and includes the Guidelines of GMP. The availability of a dedicated GMP-laboratory for the radiosynthesis and formulation of the radiotracer is therefore essential. The GMP facility ensures good quality of the final product complying with the current GMP guidelines. To support the submission of dossiers to the relevant health authorities for approval, it is mandatory that preclinical toxicological studies with the non-radioactive substance are performed prior to the first-in-human trial in order to investigate possible toxic effects of the radioligand to be evaluated. However, the risk for toxicological effects of PET imaging probes is generally considered as low as the injected amount is below the therapeutic dose. Finally, for the radiopharmaceutical to be used clinically, local authorities must give the authorization and approval for its use.

To the best of our knowledge, only two PET radiolabeled folates have so far made it to the clinic. [^18^F]AzaFol (**16**) is currently being evaluated in a multicenter first-in-human trial (ClinicalTrials.gov Identifier NTC03242993) investigating the biodistribution and FR-specific tumor detection in patients with FR-negative and FR-positive metastatic ovarian and lung cancers [86]. Although the radiochemical yield of **16** was 3–9% (0.9–2.9 GBq, decay corrected), this amount of radioactivity obtained was sufficient to allow imaging studies in at least one to two patients. For commercial productions, however, further improvement of the radiosynthesis would be needed. The results of the interim analysis of 10 of the 36 planned patients with histologically confirmed ovarian cancer (OC) and non-small cell lung cancer (NSCLC) was recently reported at the Annual Congress of the European Association of Nuclear Medicine by Meisel et al. [86]. The PET/CT imaging results showed inter- and intratumoral heterogeneity and high specificity of **16** uptake in FR-α-positive tumors in the majority of the lesions. Radiation dosimetry results with 6 patients was also reported by Gnesin et al. [87]. The results of the dosimetry study with [^18^F]AzaFol revealed the highest absorbed doses to the liver, the kidneys, the urinary bladder, and the spleen and the doses were 51.9, 45.8, 39.1 and 35.4 µGy/MBq, respectively. A mean dose of 18.0 ± 2.6 µSv/MBq corresponding to an effective dose of 5.9 ± 0.8 mSv (range 5.4–7.2 mSv), which is comparable to [^18^F]FDG, was found. Completion and publication of the complete set of the results of this promising first-in-human study with [^18^F]AzaFol is eagerly awaited. To the best of our knowledge, this is the only clinical study with a fluorine-18 labeled PET folate radiopharmaceutical reported so far in patients with metastatic ovarian and lung cancers. Since in the meantime the corresponding reduced folate derivatives, 6*R*-**20** and 6*S*-**20**, in preclinical studies have shown improved performance when compared to [^18^F]AzaFol, it might be of big interest to also translate these two reduced folate radiotracers to the clinic. The other radiolabeled folate, which is in phase 1 clinical trial not for tumor imaging but rather for differentiating chronic obstructive pulmonary disease (COPD) patients from control subjects, is [^68^Ga]Ga-EC2115 from Endocyte [88].

## 5. Conclusions

FR is a promising target for the detection of FR-positive tumor tissues. As such, a series of chemically different radiolabeled folate derivatives have been designed and evaluated for their utility to target the FR in preclinical settings. The vast majority of the synthesized and biologically evaluated radiolabeled folates did not make the complete bench-to-bedside journey as most of them did not fulfill the stringent requirements for clinical translation. Among other things, the radiolabeled folate should be synthetically easily accessible and radiochemically high yielding; the radiometal for chelation should be easily accessible, affordable and available in sufficient amounts. In addition, the biological characteristics of the radiolabeled folate should be exceptional, i.e., high and specific tumor uptake with a high tumor-to-off-target ratio. If one or more of these prerequisites are not fulfilled, the complex and expensive clinical translation may not be considered. For tumor imaging, only one folate PET tracer labeled with fluorine-18 has recently made the finish line to the clinic. The hope is that more folate-based radiopharmaceuticals with improved selectivity for FR-α over the FR-β isoform, which is overexpressed mainly on activated macrophages involved in inflammation, will be developed. The development of FR-α targeting radiolabeled folates will help to reduce the risk of false- positive results.

Finally, the supporting involvement of industrial companies and hospitals in the process of clinical translation of promising candidates is indispensable as major resources and financial support are highly needed for this costly process. Therefore, a close collaboration between universities, companies and hospitals is of major importance to bring folate-based radiopharmaceuticals from bench to the bedside, thereby bringing potential benefits to patients suffering from FR-related diseases, and especially cancers.

## Figures and Tables

**Table 1:** Chronological development of PET (positron emission tomography) radiolabeled folate tracers for folate receptor (FR)-positive tumor imaging.

Author	Year	Radiolabeled Folate	Main Findings	Ref.
Pendant Approach (FA-Based Derivatives)				
Mathis et al.	2003	α/γ-[^66^Ga]/[^68^Ga]Ga-deferoxamine-folate	High kidney and abdominal uptake	[22]
Rossin et al.	2005	^64^Cu[Cu]TETA-SCK-folate	Tumor uptake of 5.9% IA/g and high liver uptake of 33.8% IA/g at 4 h p.i. (post injection)	[23]
Bettio et al.	2006	α/γ-[^18^F]Fluorobenzylamine-folate (**1**)	High abdominal uptake; Tu (tumor): 6.56% IA/g, Ki (kidney): 40.7% IA/g at 125 min p.i.	[24]
Ross et al.	2008	γ-Click-[^18^F]fluorobutyl-folate (**2**)	High abdominal uptake; Tu: 3.13% IA/g, Ki: 16.5% IA/g at 45 min p.i.	[25]
Al Jammaz et al.	2006, 2011	[^18^F]fluorobenzene- and [^18^F]fluoropyridine-carbohydrazide-folate (**6**)/methotrexate	Tumor uptake < 6% IA/g, rapid blood clearance	[26,27]
Fani et al.	2011	[^68^Ga]Ga-DOTA folate conjugates	Tumor uptake of around 12% IA/g, high kidney uptake of 56% IA/g 60 min p.i.	[28]
Fani et al.	2012	[^68^Ga]Ga-NODAGA-folic acid (**9**) and -5,8-dideazafolic acid conjugates	FA derivative outperformed the 5,8-dideazafolic acid derivative, fast blood clearance, high kidney uptake (Tu: 16.6% IA/g, Ki: 91.5% IA/g 60 min p.i.)	[29]
Müller et al.	2012	[^152^Tb]Tb-cm09 with DOTA chelator (**10**)	Clear visualization of tumor xenografts and kidneys at 24 h p.i.	[30]
Fischer et al.	2012	γ-Click-[^18^F]fluoro-deoxy-glucose-folate (**3**)	High liver uptake; Tu: 10.0% IA/g, Ki: 42.9% IA/g at 60 min p.i.	[31]
Al Jammaz et al.	2012	[^18^F]FDG-folate/methotrexate	Tumor uptake < 4% IA/g, rapid blood clearance	[32]
Gent et al.	2013	[^18^F]Fluoro-PEG-folate (**7**)	Targeting of activated macrophages in rat model of arthritis	[33]
Fischer et al.	2013	Album-binding [^18^F]FDG-folate (**4**)	Tu: 11.5% IA/g, Ki: 13.4% IA/g at 60 min p.i.; Tu: 15.2% IA/g, Ki: 18.1% IA/g at 4 h p.i.	[34]
Kularatne et al.	2013	4-[^18^F]Fluorophenyl- and [^68^Ga]Ga-DOTA-folates	Targeting of activated macrophages in rodent inflammatory paw model	[35]
Schieferstein et al.	2013	[^18^F]Oligoethyleneglycol-folate	Tu: 3.39% IA/g; Ki: 40.8% IA/g at 60 min p.i.	[36]
Müller et al.	2013	[^44^Sc]Sc-cm09 with DOTA chelator (**11**)	Favorable tissue distribution with low uptake in non-targeted tissues Tu: 8.37% IA/g, Ki: 19.2% IA/g at 2h p.i.; Tu: 14.1% IA/g, Ki: 22.2% IA/g at 20 h p.i.	[37]
Schieferstein et al.	2014	[^18^F]-Labeled folate-pHPMA	High kidney uptake and low tumor uptake of < 0.5% IA/g at 2 and 4 h p.i.	[38]
Al Jammaz et al.	2014	[^68^Ga]Ga-NOTA- and -Ga-NOTAM-folate conjugates	Moderate uptake in the kidneys and liver; NOTA-folate: Tu: 17.8% IA/g 4 h p.i.; NOTAM-folate: Tu: 8.65% IA/g 4 h p.i.	[39]
Farkas et al.	2016	[^64^Cu]Cu- and [^68^Ga]Ga-rf42 with albumin-binding entity and NODAGA chelator (**12** and **13**)	More promising features of [^64^Cu]-rf42, high tumor-to-kidney ratio of 0.73. Tu: 13.4% IA/g, Ki: 25.3% IA/g at 2 h p.i.; Tu: 16.2% IA/g, Ki: 29.5% IA/g at 20 h p.i.	[40]
Jain et al.	2016	[^68^Ga]Ga-NOTA-folic acid	No in vivo results with FR-positive tumor-bearing mice reported	[41]
Chen et al.	2016	[^18^F]Folate-NOTA-AlF (**8**)	Low liver uptake; Tu: 10.9% IA/g, Ki: 78.6% IA/g at 90 min p.i.	[42]
Boss et al.	2016	α- and γ-Click-[^18^F]fluorobutyl-folates (**2**), -[^18^F]fluoroethyl-folates (**5**), and -[^18^F]FDG-folates (**3**)	α-isomers show significant increased kidney uptake, γ-isomers significantly higher liver uptake (see publication for biodistribution results)	[43]
Brand et al.	2017	[^68^Ga]Ga-NOTA-PEG-folate	Moderate tumor and kidney uptake, low liver uptake (<1% IA/g at 4.5 h p.i.) Tu: 6.61% IA/g, Ki: 21.7% IA/g at 4.5 h p.i.	[44]
Chen et al.	2017	Folate-PEG-NOTA-Al[^1 8^F]	Low liver uptake; Tu: 9.20% IA/g, Ki: 55.3% IA/g at 90 min p.i.	[45]
Ma et al.	2018	[^64^Cu]Cu-DOTA-FA-FI-G5·NHAc dendrimers	High liver uptake; Tu: 7.02% IA/g, Ki: 9.81% IA/g at 4 h p.i.	[46]
Kettenbach et al.	2018	[^18^F]DBCO- and [^18^F]Ala-folates	High kidney and abdominal uptake; tumor uptake < 2% IA/g at 60 min p.i.	[47]
Choi et al.	2018	[^68^Ga]Ga-HBED-CC-EDBE-folate	No biodistribution data, unfavorable PET imaging due to high abdominal uptake	[48]
Radford et al.	2019	[^55^Co]Co-cm10 and [^55^Co]Co-rf42 (**14**)	No improvement in tumor-to-kidney ratio compared to ^64^Cu-labeled derivatives, but lower liver uptake; tumor uptake is 17% IA/g at 4 h for both tracers.	[49]
Heo et al.	2019	^89^Zr[Zr]M9346A	Imaging agent for therapeutic agent IMGN853, which is currently in clinical trials; promising biological results, however, further studies needed	[50]
Cho et al. Park et al.	2016 2020	[^68^Ga]Ga(NF)HFCNP [^64^Cu]Cu-FANCFe	Specific uptake in FR-positive cells, no in vivo data available	[51] [52]
Zhang et al. Müller et al. Chen et al. Ke et al. Guo et al.	2005–2016	Pteroyl-conjugates	Low tumor and high kidney uptake	[53,54,55,56,57]
Integrated Approach (FA-Based Derivatives)				
Ross et al.	2010	2′-[^18^F]fluorofolic acid ([^18^F]FFA, **15**)	High abdominal and liver uptake; Tu: 9.37% IA/g, Ki: 46.1% IA/g at 75 min p.i.	[58]
Betzel et al.	2013	3′-aza-2′-[^18^F]fluorofolic acid ([^18^F]AzaFol, **16**)	High liver uptake; Tu: 12.6% IA/g, Ki: 57.3% IA/g at 90 min p.i.	[59]
Reduced Folate Derivatives				
Saeed et al.	2012	[^11^C]-*N*^5^,*N*^10^-methylenetetrahydrofolate (**17**)	No biological results reported	[60]
Vaitilingam et al.	2012	[^99m^Tc]Tc-DMTHF (**18**)	Prove of selectivity of the radiotracer to FR-α over FR-β	[61]
Boss et al.	2018	6*S*-α, 6*S*-γ, 6*R*-α, and 6*R*-γ-click-[^18^F]fluoroethyl-5-MTHF (6*S*-α-, 6*S*-γ-, 6*R*-α-, and 6*R*-γ-**19**)	Different uptake between *R*- and *S*-isomers in various organs including kidneys and liver. Tumor uptake between 8–11% IA/g at 60 min p.i.	[62]
Boss et al.	2019	6*R*- and 6*S*-3′-aza-2′-[^18^F]fluoro-5-MTHF (6*R*-**20** and 6*S*-**20**)	Tumor uptake of over 32% IA/g for both isomers at 3 h p.i. Different uptake between *R*- and *S*-isomers in various organs including kidney and liver.	[63]
